# Chimpanzee fathers bias their behaviour towards their offspring

**DOI:** 10.1098/rsos.160441

**Published:** 2016-11-09

**Authors:** Carson M. Murray, Margaret A. Stanton, Elizabeth V. Lonsdorf, Emily E. Wroblewski, Anne E. Pusey

**Affiliations:** 1 Center for the Advanced Study of Human Paleobiology, Department of Anthropology, The George Washington University, Washington, DC, USA; 2 Department of Psychology, Franklin and Marshall College, Lancaster, PA, USA; 3 Department of Structural Biology and Department of Microbiology and Immunology, Stanford University School of Medicine, Stanford, CA, USA; 4 Department of Evolutionary Anthropology, Duke University, Durham, NC, USA

**Keywords:** paternal care, promiscuity, chimpanzees, Gombe National Park, protection services

## Abstract

Promiscuous mating was traditionally thought to curtail paternal investment owing to the potential costs of providing care to unrelated infants. However, mounting evidence suggests that males in some promiscuous species can recognize offspring. In primates, evidence for paternal care exists in promiscuous Cercopithecines, but less is known about these patterns in other taxa. Here, we examine two hypotheses for paternal associations with lactating mothers in eastern chimpanzees (*Pan troglodytes schweinfurthii*): paternal effort, whereby males associate and interact more with their own infants, and mating effort, whereby males invest in mothers and offspring for mating privileges. We found that fathers associated more with their offspring than they did with non-kin infants, particularly early in life when infanticide risk is highest. Additionally, fathers and their infant offspring interacted more than expected. Notably, association between fathers and mother–infant pairs did not predict the probability of siring the mother's next offspring. Our results support the paternal effort, but not the mating effort hypothesis in this species. Chimpanzees are one of the most salient models for the last common ancestor between *Pan* and *Homo*, thus our results suggest that a capacity for paternal care, possibly independent of long-term mother–father bonds, existed early in hominin evolution.

## 1. Introduction

Promiscuous mating by females is thought to limit paternity certainty for males, and therefore paternal care owing to the potential cost of investing in unrelated offspring who provide no direct fitness benefit in return [1]. However, studies have now demonstrated that individuals in several promiscuous species are capable of paternal kin recognition (reviewed in [2]) and that fathers can bias their behaviour towards their offspring (e.g. bluegill sunfish: [3]; three-spined stickleback: [4]; dunnocks: [5]), including promiscuous primate fathers (baboons: [6–9]; mandrills: [10]; macaques: [11,12]; capuchins: [13]; langurs: [14]). Male associations with lactating females are often related to providing protection, with fathers defending infants from harassment and the presence of fathers associated with lower risk of infanticide (e.g. [14,15]). However, some studies have reported subtler paternal effects on offspring fitness. For example, in yellow baboons, fathers intervened on behalf of their offspring in conflicts [6], and father's presence during the immature period predicted accelerated offspring maturation [8]. Similarly, work in another baboon species found paternal effects on offspring outcomes; for example, Huchard *et al*. [9] reported that juvenile desert baboons accessed richer food patches when in close proximity to their fathers. These patterns suggest that males in some promiscuous primate species recognize their offspring and support a paternal effort hypothesis for male behaviour towards offspring and their mothers.

An alternate explanation for affiliative behaviour between males and lactating females and infants is the mating effort hypothesis, whereby males improve their future mating success with the mother by biasing their behaviour towards her and her current offspring, irrespective of their paternal relationship to that offspring [16,17]. Evidence in support of this hypothesis is mixed. A study of captive vervet monkeys reported that males interacted more often with infants when the mother was present [18], suggesting that the interactions may be geared toward currying favour with the mother rather than bestowing direct benefits to the offspring. Another study of wild Barbary macaques found evidence that males who provided care to infants subsequently had higher mating frequencies with the mother than did other males, indicating that males in this species adopt a ‘care-then-mate’ strategy [19]. A similar result was found in a recent study in chacma baboons [20]. However, other studies have failed to find a relationship between male investment in mothers and/or infants, and future mating success with mothers [21,22].

The bulk of work on close associations between males and females (i.e. ‘friendships’) and paternal behaviour in promiscuous primate species has come from Old World monkeys, making studies in other taxa important to understand the phylogenetic breadth of paternal recognition and investment. Among the Great Apes, two studies have investigated if fathers interact preferentially with their offspring and found mixed results. Rosenbaum *et al*. [23] recently demonstrated that male rank was a better predictor of male-immature interactions than paternity in wild mountain gorilla groups with multiple males. The authors suggest that, while mountain gorillas can demonstrate remarkable social flexibility in terms of the number of breeding males in a group, they lack kin recognition mechanisms. Alternately, a study in western chimpanzees (*Pan troglodytes verus*) found evidence suggesting that chimpanzee fathers biased their behaviour towards offspring. Lehmann *et al*. [24] demonstrated that fathers were more likely to play and groom with their own offspring than unrelated immatures. Additionally, while the aggression rates of all males towards mothers were lower around young infants relative to aggression rates when infants were absent, biological fathers maintained low levels of aggression longer after their infants' birth than other males. While the Lehmann *et al*. [24] study strongly suggests paternal kin recognition and care in this species, it did not consider the alternate mating effort hypothesis. Furthermore, work in other chimpanzee populations is necessary to determine how pervasive the patterns are across subspecies.

In this study, we investigate paternal and mating effort in wild eastern chimpanzees (*P.t. schweinfurthii*). All chimpanzee populations are characterized by a fission–fusion social organization in which party size and composition changes frequently over the course of the day. However, the subspecies differ in female sociality. The western subspecies is considered bisexually bonded with females as gregarious as males [25]. Conversely, while inter-site variation exists, East African females are generally less gregarious than males and mothers can spend up to 40–70% of their time alone with dependent offspring [26–28]. In the chimpanzee fission–fusion social system, individuals of both sexes are probably involved in maintaining, or at least allowing, association via shared party membership. In this study, we expect both lactating females and fathers would be incentivized to spend time together given their shared genetic interest in behaviours that benefit the offspring, but the costs of such association are theoretically more pronounced for males. While lactating females may face increased competition, aggression and exposure to stressors in groups with males (e.g. [29]), fathers may forego mating opportunities with sexually receptive females when they associate with lactating females because lactating females are less gregarious than cycling females [28]. Several studies have demonstrated that lactating mothers travel more slowly than males or females without offspring, presumably due to infant carrying costs or because dependent offspring that are walking independently travel much more slowly than adults [30–33]. Fathers may miss important male–male social interactions if they limit their travel to remain with mothers and their offspring, compared with non-fathers. These social bonds are critical to male chimpanzees, given that males participate in cooperative territorial defence, and form coalitions that function in dominance acquisition and access to fertile females (reviewed in [34]). High dominance rank positively predicts male reproductive success [35,36].

As in other primates, chimpanzee offspring mortality is highest in early infancy [37]. Infanticide in the first months of life is probably a major extrinsic factor influencing early mortality in chimpanzees. It is noteworthy that both males and females commit intra-community infanticide, though infanticide by females occurs at higher levels [38]. Female attackers have now been observed at several long-term study sites (Budongo: [39]; Kanyawara: [40]; Gombe: [41]). Pusey *et al*. [41] estimated a maximum of 30% of infant deaths in the first two years of life could be attributed to infanticide at Gombe National Park, if sudden disappearances of infants and pregnancies in which no infant was observed were included as probable infanticide. The risk of infanticide may theoretically select for biased association with fathers if they provide protection services, as observed in other species with high infanticide risk [15].

Here, we examine the behaviour of East African chimpanzee fathers around their offspring and infants not related through their matriline by integrating paternity data with 25 years of long-term behavioural data from Gombe National Park, Tanzania. First, we examined whether fathers associate more with the mothers of their own infants than with mothers of non-kin infants, particularly early in infancy when the risk of infanticide is highest. Similarly, we investigated whether infants and their fathers interacted more than expected based on demography and levels of infant–adult male interactions. Because higher association and/or rates of interactions could reflect either paternal care of the infant or male mating effort geared towards currying favour with the mother, we tested whether association patterns between fathers and mother–infant (MI) pairs predicted successful siring of the mother's subsequent offspring. These two analyses allow us to address the relative importance of paternal effort versus mating effort by fathers in this species. This study offers an important comparative perspective across chimpanzee subspecies and to other promiscuous primates, and provides insight into the evolution of bi-parental care in hominins.

## 2. Material and methods

### 2.1. Study site and subjects

Our study took place in the Kasekela community of chimpanzees at Gombe National Park, Tanzania, where the population has been under continuous study since 1960. Our study focuses on the period for which paternities have been genetically assigned (1989–2013). During the study period, the community ranged in size from 38 to 62, including 9–14 adult males and 12–25 adult females (adult age: more than or equal to 12 years old). To date, 17 males have been identified as fathers to offspring in the Kasekela community.

The long-term behavioural data are collected following two different protocols. First, during individual focal follows, Tanzanian field assistants attempt to follow a member of the community for an entire day (approx. 12 h), during which they systematically record changes in party composition. Second, during family follows, Tanzanian field assistants and expatriate researchers target a focal family (mother, infant and next youngest offspring) and record the behaviour (e.g. playing, grooming) and social partner of each focal family member at 1 min instantaneous point samples ([42,43], see [44] for a more detailed ethogram). Family follow duration over the course of the study varied from 6 h to 12 h (night nest-to-night nest) with the goal to follow each MI pair once per month. For this study, we used data from both follow types to create two complementary datasets: (i) adult male follow dataset, which contains data on adult male-MI pair association, and (ii) the family follow dataset from which infant–adult male interaction patterns can be summarized.

All data collection was non-invasive, observational in nature, and complied with the laws of Tanzania. Permission to conduct the research was granted by The Tanzania Commission of Science and Technology, The Tanzania Wildlife Research Institute and Tanzania National Parks Authority.

### 2.2. Adult male–mother associations

The adult male follow dataset contains follows of adult males who are not targeted during family follows, thus it allowed us to investigate adult male association with MI pairs. For analyses of association, the infancy of each individual of known paternity was divided into six-month bins up to the age of 3.5 years. Dyadic associations between adult males and MI pairs were calculated as the proportion of minutes each MI pair was observed with an adult male during his focal follows in a given six-month time period:

$$\frac{\text{no. minutes an MI pair was observed with a given adult male}}{\text{no. minutes the given adult male was followed}}.$$

In order to control for temporal changes in demography and community-level gregariousness, we standardized associations by z-transforming each father–MI pair proportion based on the proportion of time the adult male spent with each MI pair present in the community during the same six-month period. Furthermore, adult males were assigned to one of two kin categories: (i) fathers or (ii) non-kin. Non-kin are defined as males who are not close matrilineal relatives of the mother. Non-kin were identified based on long-term genealogical records and excluded adult maternal brothers, maternal uncles and maternal cousins of the infant. While some exclusively paternal kinship can be assigned, these data are more limited. Furthermore, the degree to which paternal kinship influences male–female associations in chimpanzees remains an open question. Adult male–infant dyads who were maternal kin, dyads including infants with unknown paternity and dyads consisting of a father with his infant of a different age were included in z-transformations. However, our analyses focused on the comparison between the association of a father with his infant and his association with non-kin infants. Maternal kin may receive inclusive fitness benefits by biasing behaviour towards related infants, and maternal kin recognition is more likely via shared association.

We used generalized linear mixed models (GLMMs) to investigate adult male association with MI pairs in two ways. First, we compared fathers' proportion of time spent with the mother of his offspring to his time spent with mothers of non-kin within each of his offspring's six-month bins (e.g. male association with the mother of his offspring from birth on 1 January 2010 through to 30 June 2010 versus male association with all other MI pairs in the same time period 1 January 2010 through to 30 June 2010). This approach allowed us to address the question of whether fathers prefer to associate with the mothers of their own offspring, relative to lactating mothers with non-kin infants in the same time period. However, because chimpanzees can give birth at any time of year and infants remain with their mothers for years, in any given six-month period mothers of non-kin may have infants of varying ages. To control for potential differences in association owing to infant age, we also compared father association with the mother of his offspring in a given age bin to his time spent with mothers of non-kin in the same age bin, but during a different time period (i.e. male association with the mother of his 0–6 month old offspring from 1 January 2010 through to 30 June 2010 versus male association with the mother of a 0–6 month old non-kin from 15 May 1998 through to 14 November 1998). This approach allowed us to ensure that any bias in association with a male's own infant during an age bin did not simply reflect a preference for infants of that age. Similar levels of male association with their own infants and with unrelated infants of the same age bin in other time periods would suggest that males are merely attracted to infants of a certain age group in general, rather than biasing behaviour based on paternity.

Adult males had to be observed for 30 h during a given six-month period to be included in all analyses of adult male associations. Additionally, only six-month periods that included associations between an adult male and both the mother of his offspring and mothers of non-kin were included for the within time-period approach. Similarly, only males who had associations with both mothers of offspring and mothers of non-kin in the same age class were included in the between time-period approach (see table 1 for sample sizes).

**Table 1.** Number of adult males and mother–infant (MI) pairs in each six-month age bin included in analyses of adult male association with MI pairs.

approach	age bin (months)	0–6	6–12	12–18	18–24	24–30	30–36	36–42
within period	no. males	15	15	14	15	15	14	13
	no. MI pairs	44	44	42	41	41	38	35
between periods	no. males	15	15	14	15	15	14	13
	no. MI pairs	49	48	47	47	45	42	41

In both models, z-transformed proportion of time spent together was the response variable, while kin type (father versus non-kin), six-month age bin, and the interaction of kin type and age bin served as fixed explanatory variables. Given previous work demonstrating that infant sex relates to maternal gregariousness [45], we tested for a main effect of infant sex on adult male associations with MI pairs; the effect was not significant and therefore not included in the final models. Father identification (ID), mother ID and infant ID were included as random effects in the models to account for repeated measures and uneven sampling. Both GLMMs were modelled using a Gaussian error distribution and identity link function. Because of our *a priori* prediction that fathers would spend more time with their offspring early in life when infants are the most vulnerable, we also conducted Tukey's pairwise comparisons between the two kin categories within each individual age bin.

### 2.3. Infant–adult male interactions

While the adult male follow dataset contains information on adult male behaviour, the family follows dataset contains great detail on infant social interactions. Therefore, to investigate patterns of infant interaction with adult males, we used family follow data to compare the observed proportion of time fathers or non-kin males spent interacting with infants to expected proportions based on community demographics. Here, infancy was divided into two age classes: (i) early: from birth to six months of age, when there is the greatest risk of infanticide and infants are in almost constant contact with the mother, and (ii) late: from six months to 3.5 years of age, when infant interactions with non-mothers are at higher levels. We pooled successive periods from six months to 3.5 years together given the generally low rates of interactions with adult males. Infants had to be observed for a minimum of 10 h in early infancy (*N*_infants_ = 23) and 60 h in late infancy (*N*_infants_ = 25) to be included in these analyses.

We calculated dyadic rates of interaction between adult males and infants as the proportion of minutes each infant spent interacting via grooming or playing with each adult male:

$$\frac{\text{no. minutes grooming or playing with a given adult male}}{\text{no. minutes observed in the same party as that adult male}}.$$

These adult male–infant interaction rates were then summed for each infant in each period to get the total strength of each infant's interactions with adult males. As above, adult male–infant dyads who were maternal kin or whose maternal kinship was unknown were included in the calculation of total dyadic interaction strength, but not in further analyses. We then determined the proportion of the total strength that was owing to interactions between the infant and their father and the proportion of total strength that was owing to interactions between the infant and non-kin males by dividing the sum of interaction rates for each kin category by the total strength. For example, if the sum of all dyadic interaction rates between an infant and non-kin males is 0.02 and the sum of all dyadic interaction rates between an infant and all adult males is 0.05, the observed proportion is 0.4. In order to control for differences in the number of adult males across time and differences in the number of adult male maternal kin of each infant, we compared these observed proportions to expected values. Expected values were based on the proportion of adult males in the community belonging to a given kin category. The greater the proportion of adult males belonging to a given kin category, the more likely the infant is to interact with that kin category. For example, if there were 10 adult males in the community on an infant's birthday and nine were non-kin, the proportion of adult male non-kin in the community would be 0.9. Observed values were compared with expected values using one-sample permutation tests with 10 000 iterations. Both sample density distributions were normal and *p*-values were two-sided.

### 2.4. Associations and subsequent paternity

To investigate whether father–mother association represents mating effort, we tested for a relationship between father–mother association patterns and the likelihood of siring the next offspring. Similar to above, association was calculated from the adult male dataset as the observed proportion of minutes each mother spent in the same party as her infant's father during the first 18 months of the infant's life because this was the period during which we observed greater association between adult males and mothers of offspring compared with adult male association with mothers of non-kin infants (see Results). Adult males had to be observed as a focal for 90 h during those 18 months in order to be included in the analysis. As above, in order to control for temporal changes in demography and community-level sociality, each father–mother proportion was z-transformed based on the proportion of time the adult male associated with all MI pairs present in the community during the same time period.

z-transformed proportions were then used as an explanatory variable in a GLMM with a binomial error distribution and logit link function to predict whether the same male was likely to sire the next offspring birthed by that mother (yes/no). We also included ordinal male rank at the time of conception in our model given the known reproductive skew towards dominant males [35]. Ordinal rank was determined based on the Elo-rating method [46] where higher scores indicate a higher position in the hierarchy. Both maternal ID and paternal ID were included as random effects to control for mothers and fathers contributing an unequal number of offspring to the analysis. For all GLMMs, we evaluated assumptions of normality and homogeneity of variance using diagnostic residual plots. All analyses were conducted in R (v. 3.2.3; R Development Core Team 2013) using the lme4 package [47] for GLMMs and DAAG package [48] for permutation tests.

## 3. Results

### 3.1. Adult male–mother association

**Figure 1.**
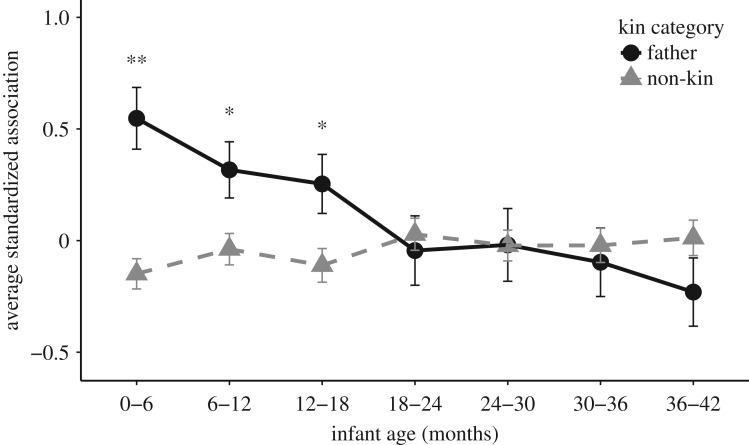
Mean ± s.e. standardized association between adult males and MI pairs in each six-month age bin by kin category for the within period approach. See table 1 for sample sizes. **p < 0.001; *p < 0.05.

When comparing adult male time spent with the mother of his own offspring versus mothers of non-kin in the same six-month period, we found a significant interaction between kin type and offspring age class (*F*_6, 1298.9_ = 4.36, *p* < 0.001). Tukey's pairwise post hoc tests revealed significant differences during age classes 0–6, 6–12 and 12–18 months with males spending a greater proportion of time with mothers of their offspring than mothers of non-kin (figure 1).

**Figure 2.**
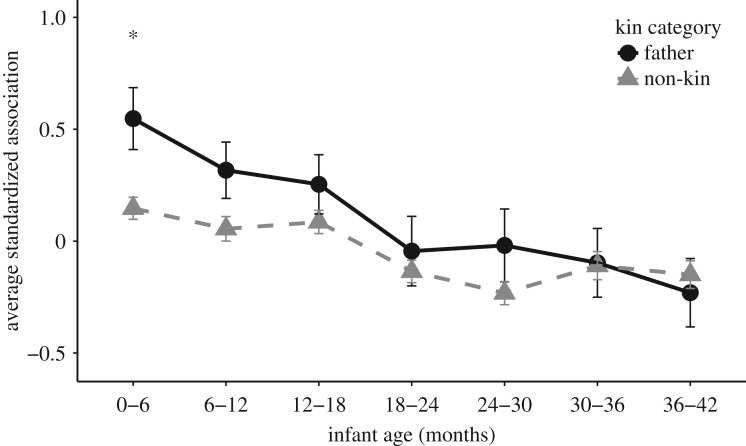
Mean ± s.e. standardized association between adult males and MI pairs in each six-month age bin by kin category for the between period approach. See table 1 for sample sizes. *p < 0.05.

When examining adult male time spent with mothers of infants of the same age class, we found that age class was a significant predictor of standardized association (*F*_6, 2118.8_ = 10.92, *p* < 0.001), while kin category tended to predict standardized association (*F*_1, 2130.5_ = 3.01, *p* = 0.083). Overall, fathers spent more time with mothers of their offspring compared with mothers of non-kin (mean ± s.e. standardized association: fathers = 0.120 ± 0.057; non-kin = −0.041 ± 0.021). The interaction between kin category and age class was not significant (*F*_6,2109.5_ = 1.178, *p* = 0.315); however, again, due to our *a priori* prediction that fathers will spend more time with offspring early in life when infants are most vulnerable, we conducted Tukey's pairwise analyses of each individual age class by kin category. These analyses revealed that fathers spent significantly more time with offspring compared with non-kin only in the 0–6 month age bin (figure 2).

### 3.2. Infant–adult male interactions

**Figure 3.**
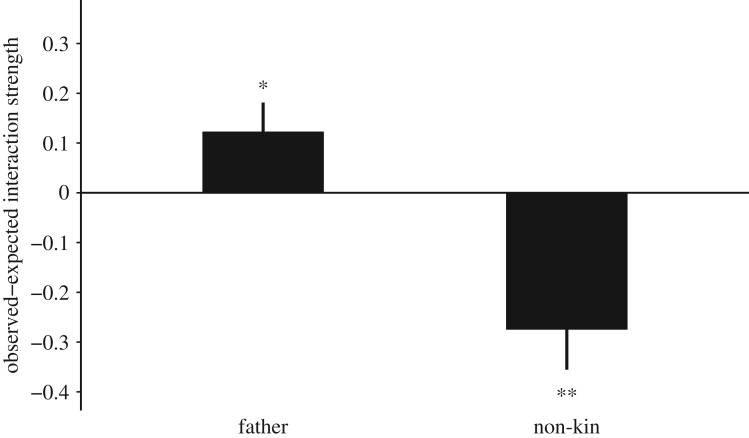
Mean ± s.e. observed minus expected proportion of time infants (*N*_infants_ = 25) during late infancy spent interacting with individuals from each kin type. **p < 0.001; *p < 0.05.

In early infancy, infants rarely interacted with adult males with only 3 min of interaction observed in this sample. In late infancy, infants spent an average 0.005 ± 0.001 s.e. proportion of time interacting with adult males overall; however, during that time infants spent a significantly greater than expected proportion of time interacting with fathers and a significantly lower than expected proportion of time interacting with adult male non-kin (figure 3).

### 3.3. Adult male–mother associations and subsequent paternity

In total, out of the 27 cases where paternity was known for subsequent offspring, the same male sired six (22%). Twenty-two cases met the minimum observation time criterion of 90 h and in six of these cases the same male sired subsequent offspring. Those cases that met the minimum observation time criterion were included in the GLMM described above. Standardized father–mother time spent together during the first 18 months of the infant's life did not predict the likelihood of the father siring the subsequent offspring (Wald *χ*^2^_1_ = 1.262, *p* = 0.261). The father's rank at conception of the subsequent offspring also did not predict the likelihood of fathering the subsequent offspring (Wald *χ*^2^_1_ = 0.022, *p* = 0.883).

## 4. Discussion

A long-held assumption is that promiscuity reduces the likelihood of paternal behaviour, as males may accidently invest in unrelated infants [1]. However, studies have now demonstrated father–offspring recognition and biased behaviour by fathers in several promiscuous species (e.g. hyenas: [49]; primates: [6]). A growing body of work in Cercopithecine primates raises the intriguing question about whether paternal kin recognition is the ancestral state in this subfamily (e.g. [50–52]), but work in other Cercopithecines and other old world primates is important to increase our understanding of these patterns at higher taxonomic levels. Here, we demonstrate biased association and interactions between fathers and their offspring, relative to infants unrelated through the matriline, yet father–mother association patterns did not translate to a higher probability of siring future offspring with the same mother. Our results therefore provide support for a paternal effort hypothesis and against a mating effort hypothesis for father–infant association and interactions in this species.

Similar to patterns reported in western chimpanzees [24], we found that fathers played with and groomed their own offspring more than expected. The consistency of these patterns across populations and subspecies suggests that some degree of paternal kin recognition is a salient feature of chimpanzees. Notably and in contrast with the Lehmann *et al*. [24] study, we also demonstrate that fathers associated more during early infancy with MI pairs for whom they were sires than with MI pairs to whom they were not related through the matriline. Here, we took the perspective of adult males and the proportion of their time spent associating with MI pairs to test how behaviour with lactating females and their infants changes with paternity; therefore, we cannot exclude the possibility that the mother may have additional male ‘friends’ as observed in other species. A study in yellow baboons, for example, found that mother–male friendships provided benefits to infants in terms of protection from harassment, but just around half of these friendships were with the genetic fathers of infants [22]. Future studies should therefore investigate the presence and potential benefits of male–female friendships beyond those of the father–mother dyad.

Interestingly, higher association between fathers and their offspring pairs waned during infancy; the father associated with the mother of his offspring at levels that are indistinguishable from his association with other lactating females by 18 months. We suggest that this early association by fathers may provide protection services. Young infants face a much higher risk of infanticide than do older infants ([41], reviewed in [38]), which may be attenuated by associating with protective males. An early study by Goodall [53] suggested that mothers associate with males after birth as a counter-infanticide strategy. The differences in association reported here may therefore explain why infanticide is not more prevalent in this species, as suggested by comparative work in other primates (reviewed in [15]). However, future work should investigate whether fathers and mothers are more likely to associate in risky situations. Such situations include border areas where inter-community encounters are more likely, or risky parties that contain infanticidal individuals [38]. A study in a Ugandan population previously demonstrated that female–female aggression rates were lower when adult males were present [54] so it seems likely the presence of less aggressive fathers [24] may mitigate female aggression that could result in infant injury or death.

It is important to note here that we cannot differentiate the relative responsibility of the father and mother for the observed association patterns. In the chimpanzee fission–fusion social system, individuals are free to join and leave groups based on the costs and benefits of conspecific interactions. This is especially true in populations without high predation risks, such as Gombe. Thus, it is likely that chimpanzee mothers and fathers both play an active role in their association. Mothers would benefit tremendously from preferential association with ‘safe’ males if males provide protection services as described above; infant mortality is one of the most critical determinants of reproductive variance among chimpanzee mothers given their long gestation period and slow reproductive rates [37]. We therefore expect that mothers should have a prime interest in associating with the father and adult male relatives. However, there is evidence to suggest that fathers also play an active role in the association. As cited above, lactating mothers travel more slowly than males or females without offspring [30–33]. Thus, males would have to adjust their travel speed to remain with mothers. Such a restriction could come at the expense of foraging effort and/or efficiency, for example.

How fathers recognize their offspring, or vice versa, remains an open question for future investigation in this species. Potential kin recognition cues include mating history with the mother and/or phenotype matching, both of which have been described in other primates (reviewed in [2]). In general, mating history is often correlated with male–infant associations in wild primates (e.g. [6,14]), but testing for phenotype matching requires a captive setting. It seems likely that both mechanisms may contribute to paternal kin recognition in chimpanzees based on previous findings in wild and captive populations. Lehmann *et al*. [24] reported that Tai females tended to copulate more with the fathers of their offspring than other males and suggested that females may use these relative copulation frequencies to assess paternity. In a series of captive studies, Parr and co-workers demonstrated that chimpanzees are able to assess kinship through facial recognition [55,56].

This is, to our knowledge, the first study to provide evidence of paternal care in eastern chimpanzees. As more evidence accumulates, it raises the possibility that all promiscuous primates have kin recognition mechanisms that could facilitate some degree of paternal behaviour, be it association for protection or more direct care. An obvious next question is how these patterns relate to offspring outcomes. Any fitness gains owing to parental behaviour provide the opportunity for natural selection to act and can inform our understanding of how and when pronounced paternal investment evolved in hominins. Paternal care is one of two main hypotheses invoked to explain the transition from promiscuity to social monogamy. The alternate hypothesis proposes that pair bonding evolved out of mate guarding when females were highly dispersed across the landscape. In this context, there remains considerable debate about whether pair bonding preceded paternal involvement in the human lineage (e.g. [57–63]). Chimpanzees represent one of the most salient models for the our shared ancestor at the *Pan*-*Hominin* split, so understanding the role of fathers in this species is a critical piece of the puzzle. Our results suggest that a capacity for paternal care existed early in hominin evolution and can occur in the absence of long-term male–female bonds.

## Data accessibility

Data are available from Dryad: http://dx.doi.org/10.5061/dryad.t8p51 [64].
